# The intracellular region of Notch ligands: does the tail make the difference?

**DOI:** 10.1186/1745-6150-2-19

**Published:** 2007-07-10

**Authors:** Alessandro Pintar, Alfredo De Biasio, Matija Popovic, Neli Ivanova, Sándor Pongor

**Affiliations:** 1 International Centre for Genetic Engineering and Biotechnology (ICGEB), Protein Structure and Bioinformatics Group, AREA Science Park, Padriciano 99, I-34012 Trieste, Italy

## Abstract

The cytoplasmic tail of Notch ligands drives endocytosis, mediates association with proteins implicated in the organization of cell-cell junctions and, through regulated intra-membrane proteolysis, is released from the membrane as a signaling fragment. We survey these findings and discuss the role of Notch ligands intracellular region in bidirectional signaling and possibly in signal modulation in mammals.

This article was reviewed by Frank Eisenhaber, L Aravind, and Eugene V. Koonin.

## Background

Notch-mediated signal transduction controls cell fate and is a key process in tissue patterning and morphogenesis [1]. Both receptors and ligands are membrane-bound proteins, the first ones being non-covalent, membrane-spanning heterodimers, the latters single pass, type I membrane proteins [1]. In response to ligand binding, the membrane-spanning subunit of the receptor (NTM) is cleaved by an extracellular ADAM-type (A Disintegrin And Metalloprotease) proteinase. This cleavage facilitates a further cleavage of NTM, within the trans-membrane region, carried out by the presenilin/γ-secretase protease and releases the intracellular domain (ICN) from the membrane [2]. This series of controlled proteolytic events is referred to as "regulated intra-membrane proteolysis" or RIP. Once translocated into the nucleus, the ICN interacts with nuclear factors that activate transcription, the main target being a transcription factor (CSL) called CBF1/RBP in mammals, Suppressor of Hairless in *Drosophila*, and LAG-1 in *C. elegans*.

The core mechanism of the Notch pathway can be thus viewed as the release of a transcriptional regulator from the membrane, triggered by ligand/receptor interactions and controlled at various levels. The established role of Notch signaling in angiogenesis [3], in T cell development [4], in the maintenance of stem cells [5], in genetic disorders [6] as well as in cancer [7,8] has been extensively reviewed. The Notch pathway has been identified as a new potential target for cancer therapy [9,10] and might also be involved in cognitive disorders [11]. Other aspects of Notch signaling, such as its regulation by endocytic processes [12] and receptor glycosylation [13], and the cross-talk between Notch and other signaling pathways [14,15] have also been reviewed.

Here, we focus on a relatively recent and potentially novel aspect of Notch signaling in mammals: the role of the intracellular region of the membrane-spanning ligands in the interaction with membrane-associated proteins, in the endocytic processes that control receptor/ligand interactions, and as membrane-tethered signaling fragments.

### Does the tail make the difference?

**Figure 1:**
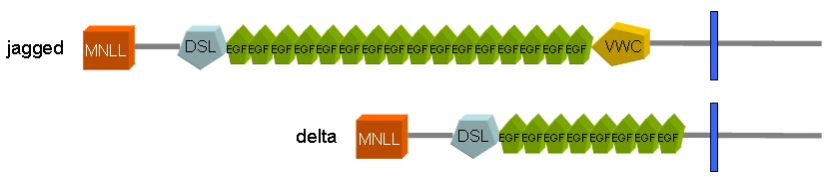
Domain architecture of Notch ligands. Typical domain organization of Notch ligands: MNLL, N-terminal domain; DSL, Delta/Serrate Ligand domain; EGF, Epidermal Growth Factor repeat; VWC, von Willebrand Factor type C domain. The transmembrane segment is shown as a blue bar. The number and type of EGF repeats can vary.

**Figure 2:**
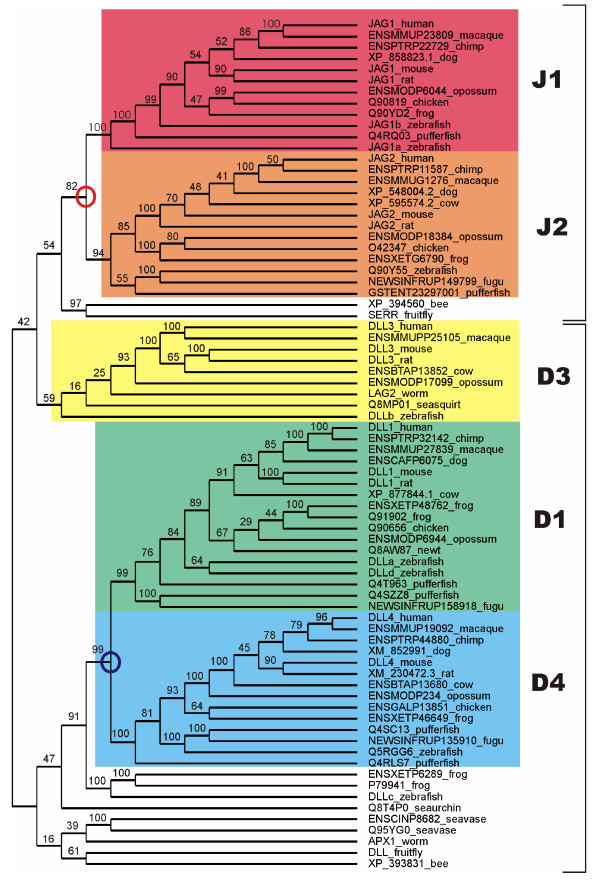
Sequence analysis. The intracellular regions of Notch ligands from different organisms were aligned automatically using ClustalW (score matrix: Gonnet, penalty for gap opening, 10; penalty for gap closing, 1; penalty for gap extension, 0.2; penalty for gap separation, 8). The cladogram was generated using the neighbor joining algorithm and drawn using Mega [41]. Confidence values for grouping in the tree were obtained by bootstrapping (N = 1000) and normalized to 100. Identified groups are labelled as J1, J2, D1, D4, D3, and colored accordingly. The branching points between J1 and J2 and between D1 and D4 groups are also labeled. Ligands sharing the same architecture in the extracellular regions are enclosed in brackets. Similar results were obtained using T-Coffee [42] and MUSCLE [43].

**Figure 3:**
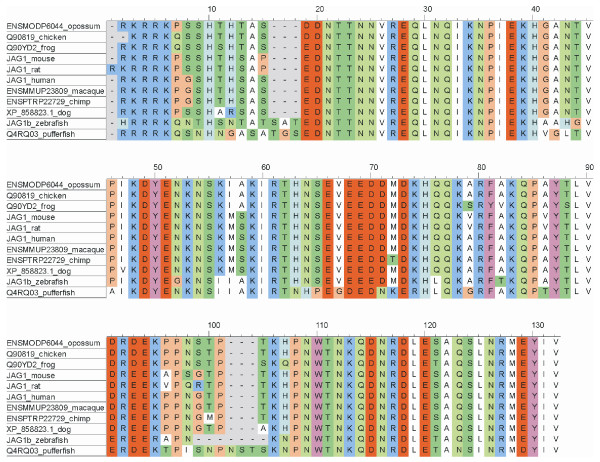
Sequence alignment, group J1. Sequences were aligned using ClustalW and colored using CINEMA. Acidic residues (D, E) in red; basic (K, R) in blue; histidines (H) in light blue; aliphatic (A, V, L, I, M) in white; small hydrophobic (G, P) in orange; aromatic (F, Y, W) in magenta; hydroxyl-containing (S, T) in dark green; amide containing (N, Q) in light green; cysteines (C) in yellow.

**Figure 4:**
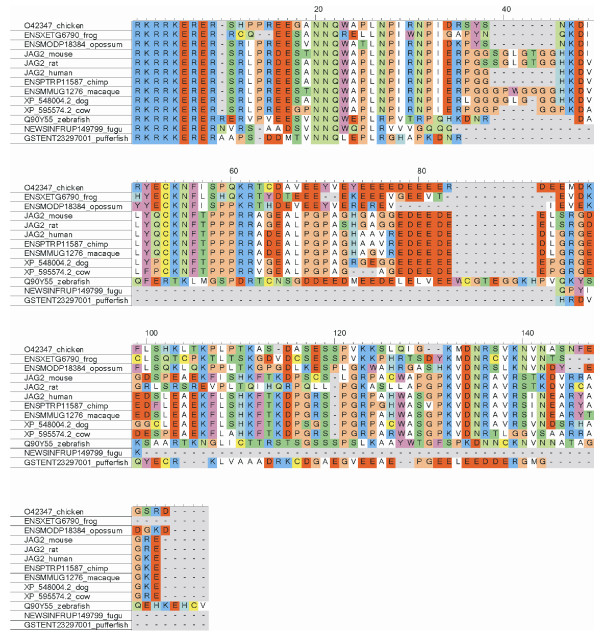
Sequence alignment, group J2. Sequences were aligned using ClustalW and colored using CINEMA. Acidic residues (D, E) in red; basic (K, R) in blue; histidines (H) in light blue; aliphatic (A, V, L, I, M) in white; small hydrophobic (G, P) in orange; aromatic (F, Y, W) in magenta; hydroxyl-containing (S, T) in dark green; amide containing (N, Q) in light green; cysteines (C) in yellow.

**Figure 5:**
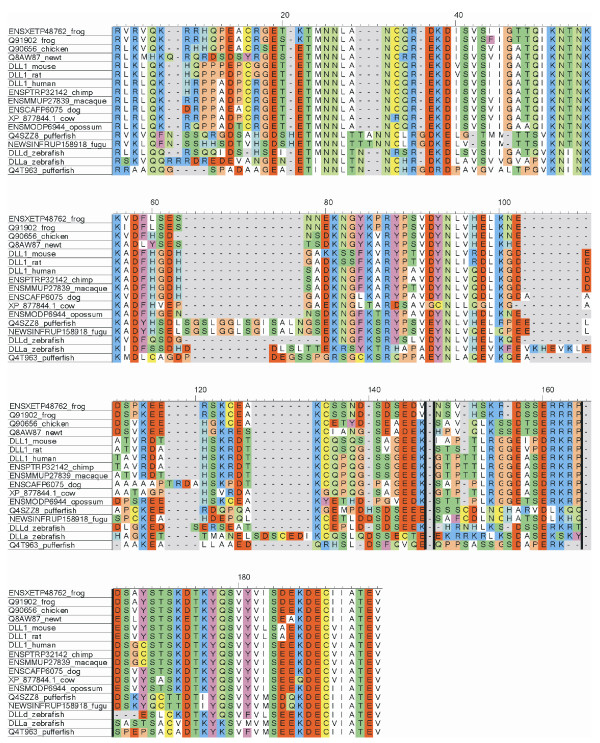
Sequence alignment, group D1. Sequences were aligned using ClustalW and colored using CINEMA. Acidic residues (D, E) in red; basic (K, R) in blue; histidines (H) in light blue; aliphatic (A, V, L, I, M) in white; small hydrophobic (G, P) in orange; aromatic (F, Y, W) in magenta; hydroxyl-containing (S, T) in dark green; amide containing (N, Q) in light green; cysteines (C) in yellow.

**Figure 6:**
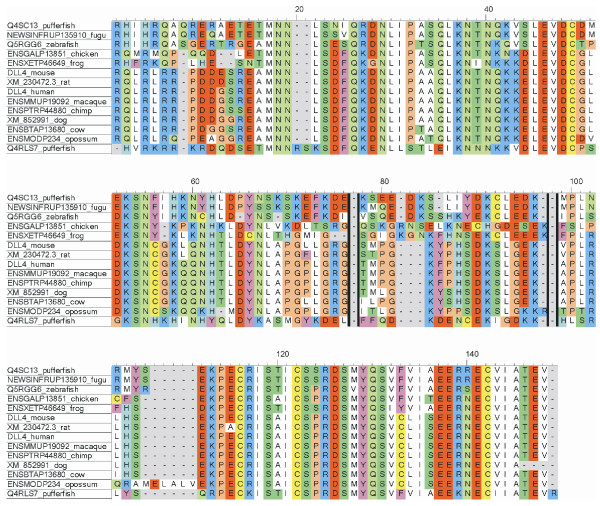
Sequence alignment, group D4. Sequences were aligned using ClustalW and colored using CINEMA. Acidic residues (D, E) in red; basic (K, R) in blue; histidines (H) in light blue; aliphatic (A, V, L, I, M) in white; small hydrophobic (G, P) in orange; aromatic (F, Y, W) in magenta; hydroxyl-containing (S, T) in dark green; amide containing (N, Q) in light green; cysteines (C) in yellow.

**Figure 7:**
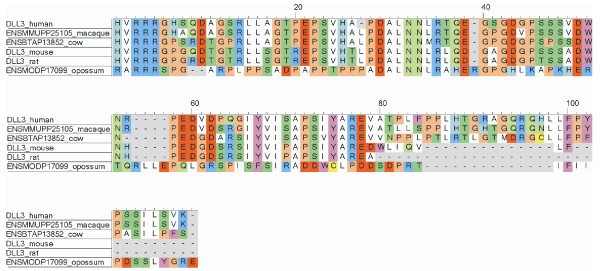
Sequence alignment, group D3. Sequences (from mammals only) were aligned using ClustalW and colored using CINEMA. Acidic residues (D, E) in red; basic (K, R) in blue; histidines (H) in light blue; aliphatic (A, V, L, I, M) in white; small hydrophobic (G, P) in orange; aromatic (F, Y, W) in magenta; hydroxyl-containing (S, T) in dark green; amide containing (N, Q) in light green; cysteines (C) in yellow.

**Figure 8:**
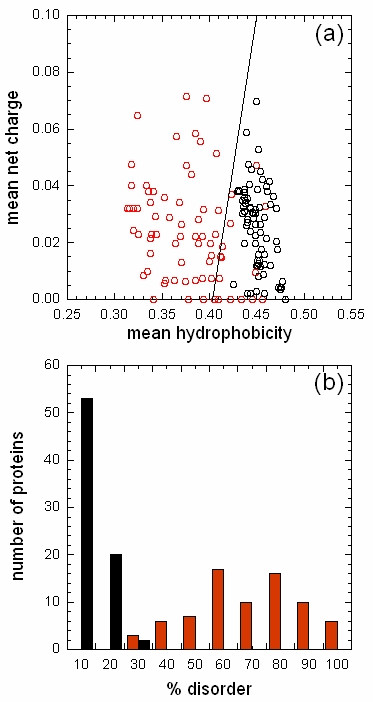
Intrinsic disorder. Disorder in the extracellular (black circles/bars) and intracellular (red circles/bars) regions of Notch ligands are shown as (a) a plot of the mean net charge v. the mean hydrophobicity [44] and (b) as the percentage of disordered residues calculated by DisEMBL using the "hot loops" definition [45]. In (a), the border between folded and natively unfolded proteins is drawn as a line.

All Notch ligands share a similar architecture (Figure 1): a poorly characterized N-terminal region required for receptor binding, a Delta/Serrate/Lag-2 (DSL) domain, a variable number of EGF-like repeats, a trans-membrane segment, and a relatively short (~100–150 amino acids) cytoplasmic tail [16]. Traditionally, ligands are classified in two distinct families: homologues of *Drosophila *Delta protein (Delta-1, -3, and -4 in mammals) and homologues of *Drosophila *Serrate (Jagged-1 and -2 in mammals). Jagged ligands have an additional, cysteine-rich region proximal to the trans-membrane segment. Within the same ligand type, the intracellular region of Notch ligands is well conserved through evolution, while different ligand types show quite distinct cytoplasmic tails. From multiple sequence alignments, the presence of relatively well distinct groups can be identified (Figure 2 and Figures 3, 4, 5, 6, 7). These groups include orthologues of human Jagged-1 (J1), of human Jagged-2 (J2), of human Delta-1 (D1) and Delta-4 (D4). Two additional, more heterogenous groups include orthologues of human Delta-3 (D3) and other more distantly related ligands. Groups J1 and J2 make a superfamily, as well as groups D1 and D4. Group D3 sits somewhat apart, and cannot be reliably assigned to any superfamily. Indeed, some of the sequences cannot be assigned to a definite group with high enough confidence. While the intracellular region of *Drosophila *Serrate does appear to be related to the J1 and J2 groups, *Drosophila *Delta is only distantly related to the D1 and D4 groups. In a similar way, *C. elegans *LAG2 can be assigned to the D3 group with low confidence and APX1 is only distantly related to the D1 and D4 groups. It can be remarked that sequence conservation is not limited to the C-terminal, PDZ-interacting motif, but extends well beyond the C-terminal residues. Predictions supported by preliminary experimental results [17] point towards a mainly disordered nature for Notch ligands cytoplasmic tail (Figure 8). On the other hand, sequence conservation within ligand types suggests that precise sequence characteristics might be required for specific patterns of post-translational modifications to take place and for specific protein-protein interactions to occur.

**Figure 9:**
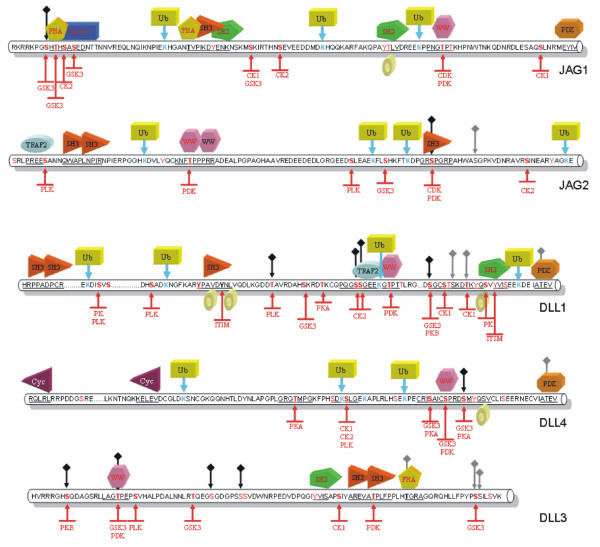
Functional analysis. Potential binding sites and post-translational modifications predicted by ELM [46, 47], NetPhos [46], and O-glycosylation [48] for the cytoplasmic tail of human Notch ligands. Prediction of ubiquitination sites is based on the preference for acidic residues adjacent to the target lysine [49]. 14-3-3, 14-3-3 proteins interacting motif (Ser/Thr phosphorylation required); Cyc, cyclin binding site; FHA, forkhead-associated domain interaction motif 1 (Thr phosphorylation required); PDZ, class I, II, or III PDZ binding motif; SH2, Src Homology 2 (SH2) domains interaction motif (tyrosine phosphorylation required; subtypes include GRB2, SH-PTP2, SRC, STAT3, STAT5, STAT6); SH3, SH3 domains binding motif (subtypes include class I, class II, and other non-canonical motifs); TRAF2, tumor necrosis factor receptor associated protein binding motif; Ub, ubiquitination site; WW, WW domain binding motif (subtypes include Group I (PPXY), Group II (PPLP), Group III, and Group IV, which requires Ser/Thr phosphorylation). Tyrosine-based sorting signals responsible for the interaction with the μ subunit of the AP (Adaptor Protein) complex are shown as doughnuts. Potential phosphorylation sites are in red; kinases are abbreviated as follows: CDK, Ser/Thr cyclin dependent kinase; CK1, casein kinase 1; CK2, casein kinase 2; GSK3, glycogen synthase kinase 3; PKA, protein kinase A; PKB, protein kinase B; PDK, Proline-Directed Kinase; PLK, Polo-like-kinase. ITIM, immunoreceptor tyrosine-based inhibitory motif (tyrosine phosphorylation required); ITSM, immunoreceptor tyrosine-based switch motif (tyrosine phosphorylation required). Sites that are candidates for O-glycosylation with β-N-acetylglucosamine are shown as grey diamonds; sites that are predicted to be both glycosylated and phosphorylated are shown as black diamonds.

**Figure 10:**
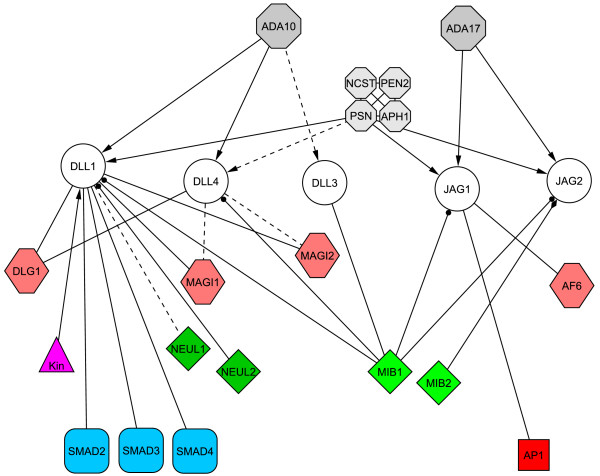
Interaction network. Summary of experimentally verified interactions for the intracellular region of human or rodent Notch ligands. Proteases are shown as octagons, PDZ-containing proteins as hexagons, E3-ubiquitin ligases as diamonds, transcription factors as smoothed squares; the AP1 enhancer element is shown as a square; phosphorylation of Delta-1 by an unknown kinase (Kin) is also shown. Interactions expected by similarity as shown as dotted lines. The graph was drawn using Cytoscape [50].

Predictions of phosphorylation, O-glycosylation with β-N-acetylglucosamine, and ubiquitination sites, as well as protein-protein interaction motifs, are shown for the cytoplasmic tail of human Notch ligands in Figure 9. It can be speculated that specific protein-protein interaction motifs on different ligands can specify different interaction patchworks. For example, PDZ-binding motifs are predicted for Jagged-1, Delta1, and Delta-4, but not for Jagged-2 and Delta-3; SH2-binding motifs are predicted for Jagged-1, Delta-1, and Delta-3, but not for Jagged-2 and Delta-4. Different phosphorylation patterns may also drive different protein interaction networks. The main experimental findings are summarized hereafter and shown in Figure 10.

### The cytoplasmic tail couples Notch ligands to PDZ-containing proteins

Independent on the interaction with receptors, the cytoplasmic tail of Notch ligands couples the Notch signal transduction machinery to PDZ containing, membrane associated proteins that play a role in the organization of cell-cell junctions. Jagged-1 has been shown to interact with the unique PDZ domain of the ras-binding protein afadin (AF6) in a PDZ-dependent manner [18,19]. Dlg1, the human homolog of the *Drosophila *Discs Large protein, was identified through peptide-affinity chromatography as a binding partner for Delta-1 and -4 [20]. It was shown that Delta-1/4 can recruit Dlg1 at cell-cell junctions, tighting cell contacts and reducing cell motility [20]. The interaction is PDZ-dependent, although it was not determined which of the three PDZ domains in Dlg1 mediates this interaction. In similar studies, the interaction between Delta-1 and members of the MAGI family (Membrane Associated Guanylate Kinases with Inverted domain arrangement) has been reported [21,22]. The interaction specifically occurs between the C-terminus of the Delta proteins and the fourth PDZ domain of MAGIs. As there are over 300 human proteins containing at least one PDZ domain [16,23], it is not clear yet whether specific recognition relies on subtle differences in the PDZ domains [24], on a binding region larger then the canonical, C-terminal PDZ-binding tetrapeptide [25], or both. It can be remarked that the C-terminus of Delta-3 and Jagged-2 do not contain any PDZ binding motif.

### Ubiquitination of the intracellular region drives endocytosis

Extensive studies on *Drosophila *and other model systems [12] have shown that the cytoplasmic tail of Notch ligands undergoes ubiquitination, which in turn drives endocytosis. As endocytosis removes the ligand from the cell surface subtracting it from the interaction with the receptor, different, non-mutually exclusive models were proposed to solve this apparent contraddiction. Ligand endocytosis would create a mechanical "pulling force" on the receptor. This force would either promote a conformational change that exposes the juxtmembrane region of the receptor to proteolytic cleavage [26], thus triggering the second cleavage of the ICN, or physically dissociates the Notch heterodimer directly promoting activation [27]. It has also been proposed that ligands would be "activated" in the acidic endosomal compartments, before being recycled to the cell surface. Alternatively, ligands would cluster in multivesicular bodies before being released to the extracellular space as exosomes. Four E3 ubiquitin ligases have been identified in mammals, Mind Bomb-1 (Mib1), Mind Bomb-2 (Mib2, Skeletrophin), Neuralized-1 (Neur1) and -2 (Neur2). Mib and Neuralized proteins display different domain architectures, which might be related to their interaction with different targets: Mibs contain a HERC2/ZZ Zinc finger/HERC2 block followed by a series of ankirin repeats and two or three RING finger domains, Neur1 contains two Neuralized domains and a single RING finger, Neur2 a Neuralized domain and a Socs box. Mouse Mib1 was shown by co-immunoprecipitation to bind all Notch ligands in HEK293A cells and to promote their endocytosis in COS7 cells [28]. Knock-out [28] or mutation [29] of the Mib1 gene lead to developmental defects and death in mouse embryos. Mouse Mib2, although functionally related to Mib1, has a different expression pattern [30] and bind and ubiquitinates specifically Jagged-2, and not the other Notch ligands [31]. Knock-out of the Neur1 gene lead to viable and morphologically normal mice, yet displaying several defects [32,33]. Finally, Neur2 was found to bind and ubiquitinate Delta-1 in HEK293A cells [34]. However, Neur2 and Mib1 displayed different subcellular localization, suggesting different and complementary roles for these two E3 ligases. Whereas in model organisms the only apparent function of the intracellular region is to carry lysine residues that can be ubiquitinylated to trigger endocytosis [35,36], it is not clear yet whether in mammals the differences in the cytoplasmic tails are underlying more specific mechanisms to control the endocytic pathways.

### Notch ligands undergo regulated intra-membrane proteolysis

It has been recently shown that in mammals Notch ligands undergo a proteolytic processing similar to that reported for *Drosophila *Delta and for Notch receptors. Murine Delta-1 was shown to be sequentially processed by an ADAM proteinase and by γ-secretase, the extracellular cleavage site being localized 10 residues N-terminal to the trans-membrane segment [37]. Also rat Jagged-1 [38] and human Jagged-2 [39] were shown to undergo the same type of proteolytic processing. ADAM 17 and ADAM 10 were identified as the proteinases involved in the ectodomain shedding of Jagged and Delta, respectively. The intra-membrane cleavage site has not been determined yet. In Jagged-1, it has been proposed to be placed at the first Val residue close to the cytoplasmic region [38]. The intracellular region of these ligands, after release from the cell membrane, was localized in the cytoplasm as well as in the nucleus [37-39].

### The intracellular region as a membrane-tethered transcriptional regulator?

The regulated intra-membrane proteolysis, followed by the release from the membrane and the localization in the nucleus, suggests a possible role of the intracellular region in transcriptional regulation. In cotransfection studies, the intracellular region of Jagged-1 was able to promote transcription of a reporter gene in COS, CHO, and HEK cells specifically through the AP1 (Activator Protein 1, p39 *jun*) enhancer element [38]. Activation by Jagged-1 is at odds with AP1 repression carried out by the intracellular domain of Notch. There is no experimental evidence, however, that the intracellular region of Notch ligands can bind DNA directly and, indeed, they do not contain any recognizable DNA binding motif. More probably, they function in combination with transcriptional complexes or specific transcription factors. Evidence in this direction is given by the interaction observed between the mouse Delta-1 intracellular region and specific Smad transcription factors (Smad-2, -3, and -4) involved in TGF-β/activin signaling [40]. Interestingly, it has been noticed that one of the PDZ-containing proteins that binds Delta-1 also interacts with Activin Type 2 receptors and Smad-3 [40].

In conclusion, there is compelling evidence that bidirectional signaling is mediated by the intracellular region of Notch ligands. While the core mechanism of signal transduction mediated by Notch receptors and their ligands has been maintained through evolution, the differentiation of ligands in higher eukaryots and the unique sequence features of their intracellular region is likely to be related to specific post-translational modifications and protein-protein interaction motifs that link the Notch signaling pathway to other signaling networks. The identification of new binding partners – at the cell membrane, in the cytoplasm and in the nucleus – as well as the characterization of the post-translational modification patterns will bring new insights into this aspect of the Notch network.

## Reviewers comments

### Reviewer's report 1

Frank Eisenhaber, Bioinformatics Group, Institute of Molecular Pathology, Vienna, Austria

The authors present a review on the functional significance and the sequence pattern-function correlations within the intracellular part of Notch ligands. Whereas this review is of considerable interest, the authors might consider the following points for making their MS even more informative:

1) It would be good for the reader to see diagrams of the sequence architecture of representatives of the several Notch ligand classes as a figure.

2) There are many methods to evaluate and to predict intrinsically unfolded regions. The authors might wish to show to which extent segments of the notch ligand sequences do represent such reasons.

3) There are no Ying-Yang sites (except you define them, see page 4 bottom); better, speak about O-glycosylation sites.

4) The work would win from a more distinct summary with the conclusions explicitely listed.

#### Authors' response

1) A diagram showing the typical domain architecture of Notch ligands has been added as Figure 1.

2) Intrinsic disorder in the cytoplasmic region of Notch ligands has been calculated using two different methods. The first is based on the plot of the mean net charge v. the mean hydrophobicity, as described by Uversky *et al*. in ref. [44]; the second is based on DisEMBL (ref. [45]). The two methods are somewhat complementary, in that the first is based only on the amino acid composition and the physical properties of amino acid types, the second on the secondary and tertiary structure determinants in the sequence. Results have been summarized in a new figure (Figure 4a–b).

3) All potential O-glycosylation sites have now been included, independently on phosphorylation, and added in Figure 5, which has been revised accordingly.

4) Biology Direct allows only for a very short Abstract/Summary in "review" papers. Instead, we tried to list the main points in the "conclusion" paragraph.

### Reviewer's report 2

L Aravind, Computational Biology Branch, NCBI, NLM, NIH, Bethesda, USA

The paper reviews the role of the Notch family ligands in signal transduction. While the role of the notch intracellular regions has been intensely investigated, the role of the intracellular portions of the corresponding ligands is less understood. In this paper Pintar et al review the current understanding of the signaling functions of the cytoplasmic tails of the DSL ligands.

The key points I have are:

1) Figure 3 shows potential modification and binding sites on the human notch ligand. Given that alignments were made for the various ligand families it will be useful to prioritize the predicted binding and PTM sites based on their conservation within a ligand family. This might indicate their greater generality in terms of a conserved signaling role.

2) "The intracellular region as a membrane-tethered transcription factor?": pg 7 of the PDF file. Is the evidence really favoring the intracellular tail as a TF itself? It is better to state that the intracellular region of the DSL ligands might function in conjunction with transcription factors. The authors might want to point out that the tail itself does not seem to have any recognizable DNA binding domains and might instead function in conjunction with a known transcription factor like SMAD.

#### Minor

Stylistic issue: As per BMC specifications I believe figure 2A, 2B etc need to be split up into separate figures.

"Activation by Jagged-1 is at odd with AP1 repression..."

Activation by Jagged-1 is at *odds* with AP1 repression

Page 6: The Ub E3 ligases: you might want to specify that they have RING finger domains as the active E3 component. It would also be nice if you mentioned the interesting complex domain architectures of the Mind bomb E3s in the text (or may be show it in the fig. 4)

#### Authors' response

1) This is an interesting observation. There are actually post-translational modifications (PTM)/bindinf motifs that are consistently predicted for all species, like the PDZ binding motifs in Jagged-1 and DLL1/DLL4, and others that are less conserved. Totally conserved motifs are likely to be strictly required for fundamental processes, whereas less conserved PTMs/binding motifs may play a role in some sort of fine tuning of the developmental processes governed by Notch signaling, and might be different in different species. From a practical point of view, it is difficult to summarize all the predictions for all species, and this is why we restricted the results to human ligands. For a reader interested in a particular PTM/binding motif, it is probably easier to jump from Figure 9 to Figures 3, 4, 5, 6, 7 to check if that feature is conserved, to what extent, and in which species.

2) We agree with the reviewer's comment. The title of the paragraph has been reformulated and we specified in the text that the intracellular region of Notch ligands may play a role in transcriptional regulation, but in an indirect manner.

Figure 2 has been splitted in separate figures.

The spelling mistake has been corrected.

E3 ubiquitin ligases: we added a short paragraph mentioning the different domain architecture of these E3 ubiquitin ligases.

### Reviewer's report 3

Eugene V. Koonin, NCBI, NLM, NIH, Bethesda, USA

This is a very concise, to the point review emphasizing the diverse functions of the cytoplasmic tails of Notch ligands. The ever-growing evidence of the importance of RIP for diverse processes and the complexity of the system are remarkable.
